# Components of the *Arabidopsis* nuclear pore complex play multiple diverse roles in control of plant growth

**DOI:** 10.1093/jxb/eru346

**Published:** 2014-08-27

**Authors:** Geraint Parry

**Affiliations:** Institute of Integrative Biology, University of Liverpool, Crown Street, Liverpool L69 7ZB, UK

**Keywords:** Flowering time, nuclear pore, nuclear pore complex (NPC), nuclear transport, nucleoporin, nucleus.

## Abstract

The nuclear pore complex (NPC) is a multisubunit protein conglomerate that facilitates movement of RNA and protein between the nucleus and cytoplasm. Relatively little is known regarding the influence of the *Arabidopsis* NPC on growth and development. Seedling development, flowering time, nuclear morphology, mRNA accumulation, and gene expression changes in *Arabidopsis* nucleoporin mutants were investigated. Nuclear export of mRNA is differentially affected in plants with defects in nucleoporins that lie in different NPC subcomplexes. This study reveals differences in the manner by which nucleoporins alter molecular and plant growth phenotypes, suggesting that nuclear pore subcomplexes play distinct roles in nuclear transport and reveal a possible feedback relationship between the expression of genes involved in nuclear transport.

## Summary

Components of the nuclear pore complex have differential effects on pleiotropic growth phenotypes, mRNA transport, and gene expression, presented in a comparative study of nucleoporin function in *Arabidopsi*s.

## Introduction

The nuclear pore complex (NPC) is a massive macromolecular conglomerate that sits within invaginations of the nuclear envelope and controls nucleo-cytoplasmic transport of RNA and protein (Raices and D’Angelo, 2012). Proteomic and microscopic analysis have deciphered that the entire NPC is comprised of distinct subcomplexes that are octagonally arranged around a central channel (Cronshaw *et al.*, 2002; Alber *et al*., 2007*a*, *b*; Fiserova *et al.*, 2009; Degrasse and Devos, 2010; Tamura *et al.*, 2010). The size of a single NPC varies between ~60 MDa in yeast and ~120 MDa in metazoans but contain similar sets of core proteins. NPC subcomplexes are comprised of individual nucleoporin (NUP) proteins. These NUPs play roles in maintaining the structural integrity of the NPC (Walther *et al.*, 2003), directly influencing gene expression (Capelson *et al.*, 2010; Kalverda *et al.*, 2010; Van de Vosse *et al.*, 2013), controlling differentiation (D’Angelo *et al.*, 2012), maintaining regions of chromatin exclusion (Krull *et al.*, 2010), or modulating nuclear transport (Walde and Kehlenbach, 2010).

Over the past 15 years much of the work aimed at elucidating the nature and function of the NPC has taken place in *Saccharomyces cerevisiae* and in metazoan cell culture. However, a growing portfolio of research in *Drosophila* (Capelson *et al.*, 2010; Kalverda *et al.*, 2010), *Caenorhabditis elegans* (Galy *et al.*, 2003; Rodenas *et al.*, 2012), fission yeast (Bai *et al.*, 2004), and trypanosomes (DeGrasse *et al.*, 2009) now indicates that general NPC and specific NUP function may vary significantly between eukaryotes.

The plant NPC remained somewhat of a mystery until recent studies using proteomics (Tamura *et al.*, 2010), electron microscopy (Fiserova *et al.*, 2009), and bioinformatics (Neumann *et al*., 2006, 2010) determined that the structure of the plant NPC is probably similar to that observed in other eukaryotes. The proteomic study of Tamura and co-workers (Tamura *et al.*, 2010) demonstrated that the composition of the plant NPC is more closely aligned to the metazoan NPC than the yeast NPC. In that study, the NUPs that were identified indicate that the broad composition of the NPC subcomplexes is maintained (Tamura *et al.*, 2010). In addition, a plant-specific nucleoporin, NUP136, was identified, and suggests that in future, other plant-specific NUPs will be discovered (Tamura *et al.*, 2010).

In addition to this information about the composition of the NPC, it has been found that certain NUPs from *Arabidopsis*, tobacco, and *Lotus* are involved in diverse signalling pathways. These studies show that the NPC is a control point for the interaction of the plants with both pathogenic (Zhang and Li, 2005; Cheng *et al.*, 2009; Wiermer *et al.*, 2012) and symbiotic microorganisms (Kanamori *et al.*, 2006; Saito *et al.*, 2007; Groth *et al.*, 2010). In addition, plants with defects in NUP function have altered responses to hormone signalling (Parry *et al.*, 2006; Robles *et al.*, 2012) and abiotic stresses (Dong *et al*., 2006*a*, *b*; Lazaro *et al.*, 2012; MacGregor *et al.*, 2013). A pattern is emerging that places the NPC as a participant in many signalling cellular pathways even though the molecules whose nucleo-cytoplasmic transport is controlled by different NUPs are largely unknown (Parry, 2013).

This study presents phenotypic and molecular analysis of a range of *Arabidopsis* NUP mutants. As evidence emerges from other experimental systems that individual NUPs play specific cellular roles, it appears that NUPs influence plant growth by different molecular mechanisms. In addition, it is shown that in certain *nup* mutants, expression of genes involved in nuclear transport is up-regulated, suggesting a mechanism of feedback control.

## Materials and methods

### Growth conditions

Seedlings were grown at 22 °C for 16h light [termed long days (LDs)] or 12h light [termed short days (SDs)] on 1% agar plates with 1% sucrose, half-strength Murashige and Skoog (MS) salts pH 6, and germinated after 3 d at 4 °C. Identities of SALK T-DNA insertion lines (Alonso *et al.*, 2003) are outlined in Supplementary Table S1 available at *JXB* online. The primers used for identification of homozygous insertion lines are shown in Supplementary Table S2.

### RNA extraction and real-time PCR

After growth for 7 d in LD conditions, RNA was extracted from 100mg of seedling tissue using the Spectrum RNA kit (Sigma-Aldrich). A 1–2 μg aliquot of RNA was used to produce cDNA with a Superscript III (Life Technologies) or cDNA synthesis kit (Bioline). Non-quantitative PCR for mutant analysis was performed using Red-Hot Taq (Bioline). Real-time PCR was performed with SYBR-Green, Platinum Taq (Life Technologies), using primers for gene expression shown in Supplementary Table 2 at *JXB* online on an MJ Research Opticon 2 machine with Opticon Monitor 3 software. Quantification of expression was determined from ≥3 experiments and the values derived using the comparative C_T_ method (2^–ΔΔCt^) (Schmittgen and Livak, 2008) with ACTIN7 (At5g09810) as the internal control. Control genes At4g33060 and At3g10040 were selected at random from a data set produced from study of the *Arabidopsis* hypoxia response (Licausi *et al.*, 2011). From the microarray data described in Fig. 6, the fold change in log_2_ expression compared with Col-0 in At4g33060 is 0.054 (*nup160-1*) or 0.048 (*nup62-2*) and in At3g10040 is 0.144 (*nup160-1*) or 0.104 (*nup62-2*).

### mRNA localization

Seven-day-old seedlings were prepared similarly to previous methods (Parry *et al.*, 2006) with modifications. Briefly, seedlings were incubated with slow shaking at room temperature in glass universals with Buffer 1 [120mM NaCl, 7mM Na_2_HPO_4_, 3mM Na_2_PO_4_, 2.7mM KCl. 80mM EGTA, 0.1% Tween, 10% dimethylsulpoxide (DMSO)]+formaldehyde (5%, PFA) for >30min followed by 5min washes in 100% methanol (2×), 100% ethanol (2×), and 1:1 Buffer 1:methanol before post-fixing in Buffer 1+PFA for >30min. Samples were washed for 5min in Buffer 1 (2×) and Perfect Hyb Plus Buffer (Sigma-Aldrich), the latter in a 12-well plastic tissue culture dish. Seedlings were pre-hydridized at 50 °C in fresh Perfect Hyb Plus Buffer for >1h before addition of 0.6 pmol μl^–1^ 25-mer oligo(dT) primers tagged with fluorescein and incubated overnight. Samples were then washed with 2× SSC, 0.1% SDS and 0.2× SSC, 0.1% SDS for 1h and >20min, respectively. For visualization, samples were mounted either in propidium podide (PI; 1 μg ml^–1^) with Vectashield (Vector lab) or in Vectashleld+4’,6-diamidino-2-phenylindole (DAPI; 1.5 μg ml^–1^) and viewed using the fluorescein isothiocyanate (FITC) filter on a Zeiss Axioskop 2plus [the position of the nuclei was determined using either a tetramethylrhodamine isothiocyanate (TRITC) filter to view PI-stained nuclei or a UV filter to view DAPI-stained nuclei]. For quantification, ImageJ was used to compare pixel intensity between the nucleus and cytoplasm within individual cells and expressed as a ratio where a higher value represented higher nuclear expression, as detailed in Supplementary Fig. S5 at *JXB* online. The EVOS XL Imaging system was used (Life Technologies) to visualize ×10 images of roots.

### Measuring nuclear morphology

Seedlings were prepared as in the mRNA localization experiments except that roots were mounted in PI (1 μg ml^–1^) with Vectashield (Vector lab) or in Vectashield+DAPI (1.5 μg ml^–1^) and visualized using the TRITC (PI) or UV (DAPI) filter on the Axioskop 2plus. ImageJ was used to measure the nuclear circularity and nuclear perimeter.

### Microarray expression

RNA was extracted from three independent sets of 7-day-old wild-type, *nup62-2*, and *nup160-1* seedlings using the Spectrum Plant RNA kit (Sigma-Aldrich). A 1 μg aliquot of RNA from each sample (triplicate for each genotype) was sent to the NASCarrays facility (http://affy.arabidopsis.info) where it was prepared, processed, and hybridized to the Affymetrix AraGene-1_0-st-v1 chip using their established protocols. The resulting data was converted using R/Bioconductor2.12 (http://www.bioconductor.org) to CSV files following normalization using the GCRMA protocol. The triplicated expression values were averaged and this value from each mutant was compared with wild type. The gene ID of each sample whose expression has altered 2-fold was determined by comparison with an appropriate probe set [AraGene-1_0-st-v1.na32.tair10.probeset (version 1)] using a Perl script kindly supplied by Ben Wareham (University of Liverpool). The entire data set can be downloaded via the Iplant collaborative: http://data.iplantcollaborative.org/quickshare/aad60c79af44e93a/Exp654.zip (last accessed 4 August 2014).

## Results

### Nucleoporin mutants exhibit a range of phenotypes

A comprehensive analysis of the composition of the plant NPC revealed that most of the known metazoan and yeast NPC components could be identified in *Arabidopsis* (Tamura *et al.*, 2010), However, the contribution of many of these components to plant growth and development is unknown, while those plants with mutations in NUP genes have been investigated as part of isolated studies. Therefore, a direct comparative study of insertion mutants within many of the NUP genes was performed. This involved investigating general growth responses such as flowering time and root elongation, changes in nuclear morphology and nuclear mRNA transport, as well as global alterations in gene expression. The aim was to ascertain how alterations in the function of the nucleus might correspond to changes in plant growth. Viable homozygous mutants were selected from across NPC subcomplexes that each contribute to the different proposed functions of the NPC, as suggested from studies in other eukaryotes.

**Fig. 1.**
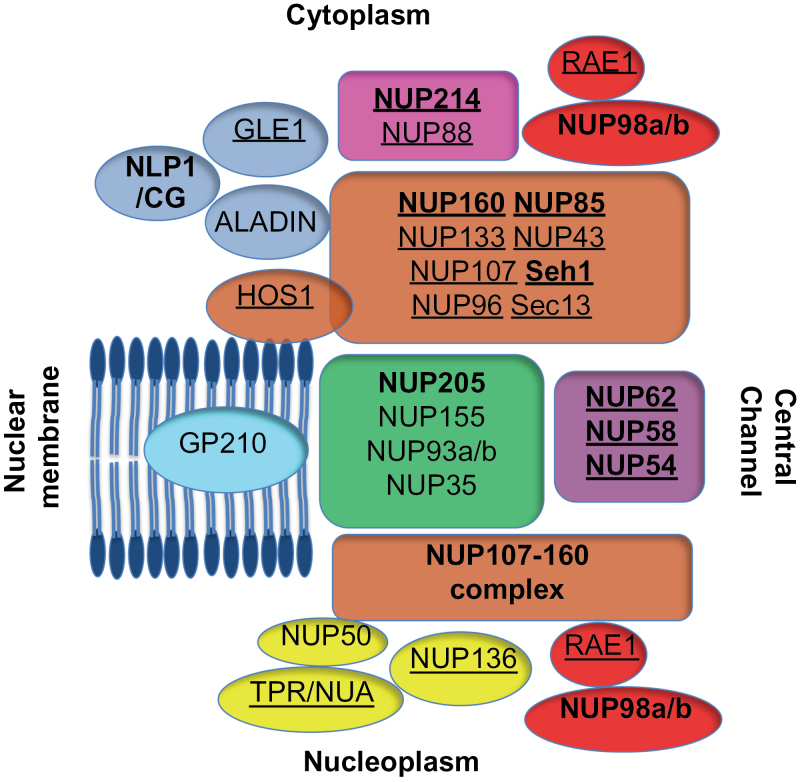
The *Arabidopsis* nuclear pore complex. Schematic of putative *Arabidopsis* nucleoporins identified during a previous proteomic screen. Nuclear pore subcomplexes are designated in colours taken from that study (Tamura *et al.*, 2010). Nucleoporins in bold are studied herein and the mutant phenotype of nucleoporins whose name is underlined have been previously published: NUP214, GLE1 (Braud *et al.*, 2012), NUP88 (Cheng *et al.*, 2009), RAE1 (Lee *et al.*, 2009), NUP160 (Dong *et al.*, 2006*b*; Parry *et al.*, 2006; Robles *et al.*, 2012), HOS1 (Dong *et al.*, 2006*a*; Lazaro *et al.*, 2012; Jung *et al.*, 2013; MacGregor *et al.*, 2013), NUP133, NUP107, NUP85, NUP43, Seh1, Sec13 (Wiermer *et al.*, 2012), NUP96 (Zhang and Li, 2005; Parry *et al.*, 2006), NUP62 (Zhao and Meier, 2011), NUP136/NUP1 (Lu *et al.*, 2010; Tamura *et al.*, 2010), and TPR/NUA (Jacob *et al.*, 2007; Xu *et al.*, 2007*b*).

Figure 1 shows the proposed arrangement of the plant NUPs into their putative subcomplexes, based on analogy to yeast and metazoan NUPs. In earlier studies, certain null *nup* mutants were identified as exhibiting embryo lethality in *Arabidopsis*, including MOS7/NUP88 (Cheng *et al.*, 2009), NUP214/LNO1, GLE1 (Braud *et al.*, 2012), NUP1/NUP136 (Lu *et al.*, 2010), *nup62-3* and *nup205-1* (Meinke *et al.*, 2008). Therefore, it was not unexpected to be unable to isolate homozygous mutants in new alleles of NUP214 (*nup214-3*, Sail_220_H11), NUP205 (*nup205-2*, Sail_874_A02), and an allele of NLP1/CG (*nlp1-1* Salk_006526) (Supplementary Table S1 at *JXB* online).

**Fig. 2.**
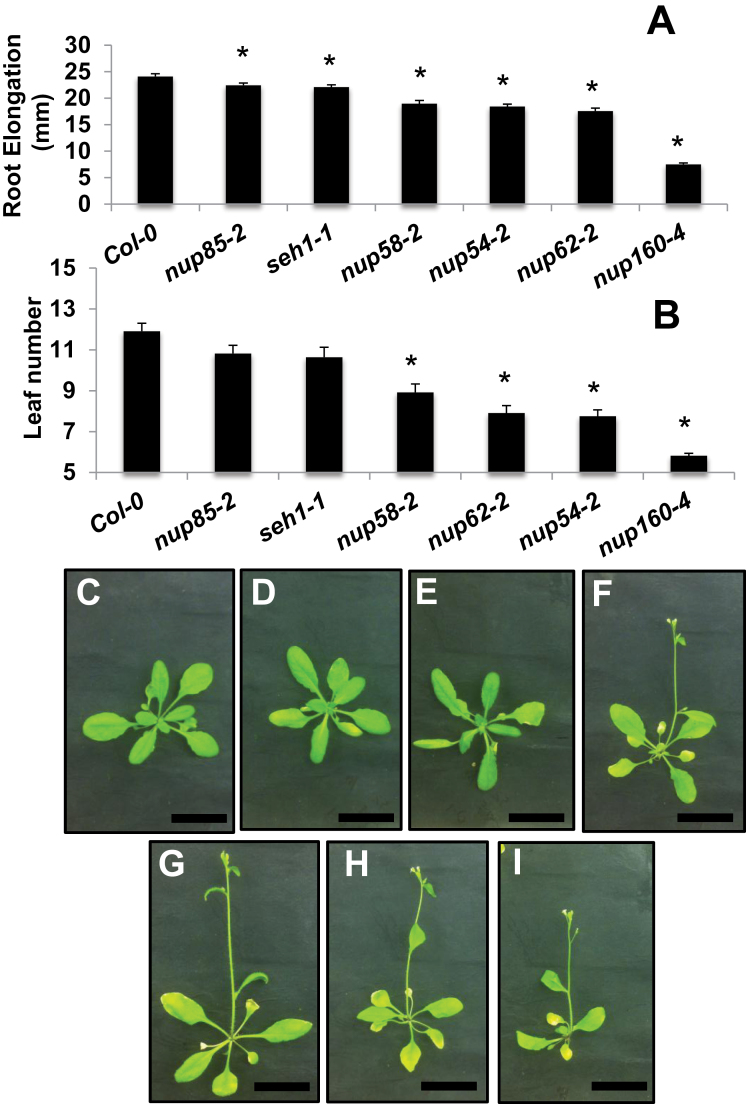
Growth changes in *Arabidopsis nup* mutants. Root elongation (A) of 7-day-old seedlings grown in LD conditions (*n*=21–46). Rosette leaf number (B) at the time of flowering of plants grown in LD conditions (*n*≥8). Error bars represent the SE. Student *t*-test, compared with Col-0, *P*<0.02(*). Representative pictures of 25-day-old plants grown under LD conditions, Col-0 (C), *nup85-2* (D), *seh1-1* (E), *nup62-2* (F), *nup54-2* (G), *nup58-2* (H), and *nup160-4* (I). The scale bar represents 3cm.

The proposed NUP62 subcomplex comprises the NUP62, NUP58, and NUP54 proteins and is thought to reside in the central pore of the NPC (Fig. 1) (Solmaz *et al.*, 2011). In this study, another lethal NUP62 allele (*nup62-4* Salk_071521) was identified and the *nup62-2* (Sail_127_F01) and *nup62-1* (Salk_037337) alleles were investigated. These plants are viable although significantly smaller in stature compared with wild-type plants (Fig. 2; Supplementary Fig. 3 at *JXB* online). *nup62-2* plants contain a T-DNA insertion at position 2061bp of the cDNA, causing a premature stop codon that presumably creates a truncated protein lacking 53 C-terminal amino acids. *Nup62-1* plants have a T-DNA insertion at the border of the fifth exon and fifth intron (equivalent to the position of amino acid 612), introducing a stop codon after an additional 47 amino acids.

The *nup54-2* (Salk_015252) and *nup58-2* (Salk_099638) mutant alleles share a similar growth phenotype to *nup62-2* reminiscent of previously identified *nup* mutants, characterized by a reduction in root elongation, a decrease in stature, and early flowering, when grown in both in LD (Fig. 2; Supplementary Fig. S3 at *JXB* online) and SD conditions (Supplementary Fig. S2). The *nup54-2* and *nup58-2* alleles do not produce a full-length transcript yet have 5′ and 3′ mRNA expression, indicating that these alleles, like *nup62-2*, probably form truncated proteins. It remains to be determined how these truncated proteins specifically impact nuclear transport in *Arabidopsis*. A structural analysis of the rat NUP62 subcomplex indicates that NUP54 individually binds both NUP62 and NUP58 to form the intact NUP62 complex (Solmaz *et al.*, 2011). These authors show that NUP62 and NUP54 interact by their N-terminal domains, so it is feasible that the truncated NUP62 and NUP54 proteins are still able to make this interaction in *Arabidopsis*. The *nup54*, *nup58*, and *nup62* mutant alleles were designated in a recent study that characterized vegetative phenotypes of these alleles (Ferrandez-Ayela *et al.*, 2013). Interestingly double mutant combinations of *nup58nup62* and *nup58nup54* alleles are viable and do not have a significantly more severe phenotype than single mutant plants.

The NUP107–160 subcomplex is predicted to play a structural role in the NPC. It is the largest NPC subcomplex comprising eight members including NUP85, NUP96, NUP160, and SEH1 (Fig. 1) and has been characterized as playing a role in the auxin and defence responses (Zhang and Li, 2005; Parry *et al.*, 2006; Wiermer *et al.*, 2012). New alleles of NUP85 (*nup85-2*, Salk_133369 and *nup85-3*, Salk_131200) were identified, both of which show a wild-type growth phenotype (Fig. 2; Supplementary Fig. S4 at *JXB* online). The T-DNA insertion in *nup85-2* is located in the 5′ part of the gene so probably causes a null mutation, but the insertion in *nup85-3* causes introduction of a premature stop codon after amino acid 477. Interestingly, *nup85* and *seh1* mutants do not share the strong *nup160-4* (Salk_126801) or *nup96* mutant phenotypes (Fig. 2; Parry *et al.*, 2006).

Therefore, the phenotypes observed in the viable *nup* mutant plants appear to fall into two broad classes: those whose growth is similar to that of wild-type plants and those that display a consistent altered growth phenotype (Supplementary Table S1 at *JXB* online).

### The NPC influences nuclear morphology

**Fig. 3.**
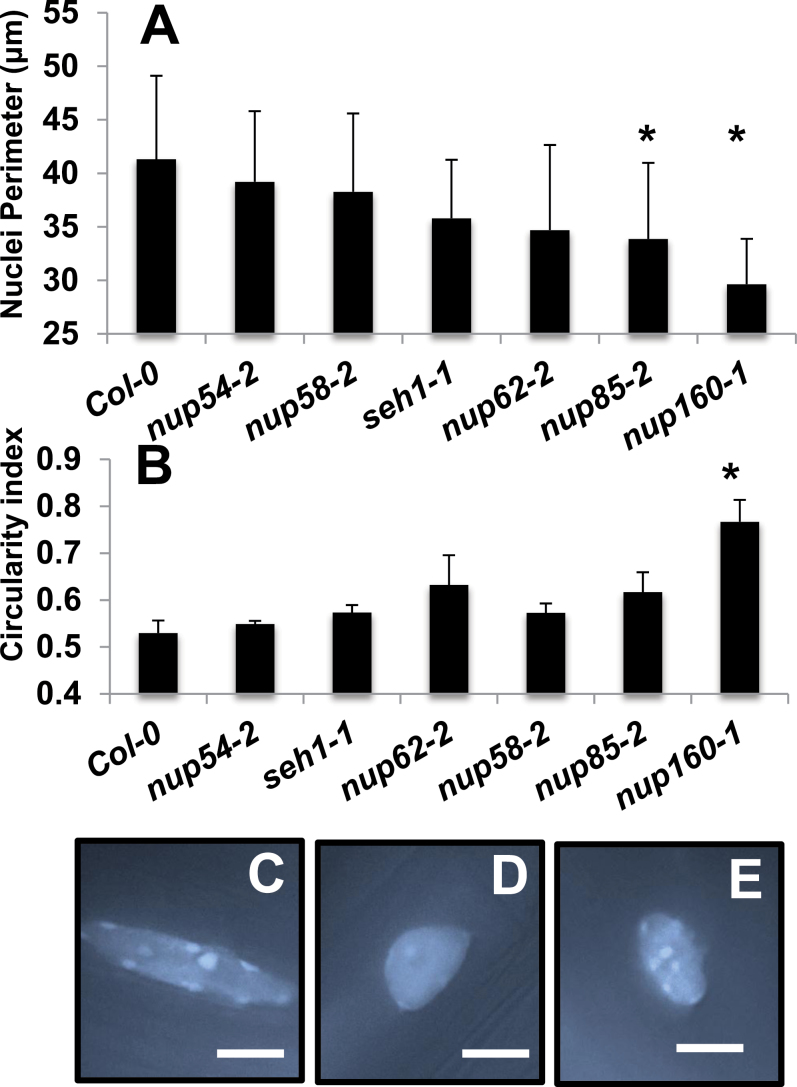
Nuclear morphology is altered in nucleoporin mutants. Nuclei perimeter size (A) or circularity (B) were measured from mature root cells of 7-day-old seedlings. In (B), a lower value represents less circular nuclei. Bars represent the SE from three independent experiments, with at least 29 nuclei per genotype. Average Student *t*-test values of original values compared with Col-0, *P*<0.01(*). Representative pictures of nuclei from Col-0 (C), *nup160-1* (D), or *sun1sun2KD* (E; Zhou *et al.*, 2012) root cells. The average circularity of *sun1sun2KD* nuclei is 0.66, *n*=47. Nuclei are stained using vectastain+DAPI (1.5 μg ml^–1^). Scale bar=10 μm.

The size and shape of plant nuclei are affected by alterations in NPC and nuclear envelope composition (Tamura *et al.*, 2010; Tamura and Hara-Nishimura, 2011; Zhou *et al.*, 2012). Nuclei from mature root cells across the range of *nup* mutants were visualized (Fig. 3) and it was found that certain mutant nuclei have a smaller nuclear perimeter. Whereas in wild-type root cells, nuclei in this cell type have an elongated morphology, the *nup160-1* [which has the same growth phenotype as *nup160-4* (Parry *et al.*, 2006)] mutant displays significantly more circular nuclei in common with *sun1KOsun2KD* mutant nuclei (Fig. 3B–E) (Zhou *et al.*, 2012). This suggests that the function of the NPC has a more profound role in controlling nuclear morphology rather than purely size. Interestingly, although other *nup* mutant plants such as *nup62*, *nup58-2*, and *nup54-2* have reduced size and are early flowering (Fig. 2), the nuclear morphology of these plants is not significantly different from that of wild-type plants. This suggests that alterations in nuclear morphology do not completely correlate with the broader *nup* mutant growth phenotypes.

### Nucleoporins play differing roles in the control of mRNA nuclear export

**Fig. 4.**
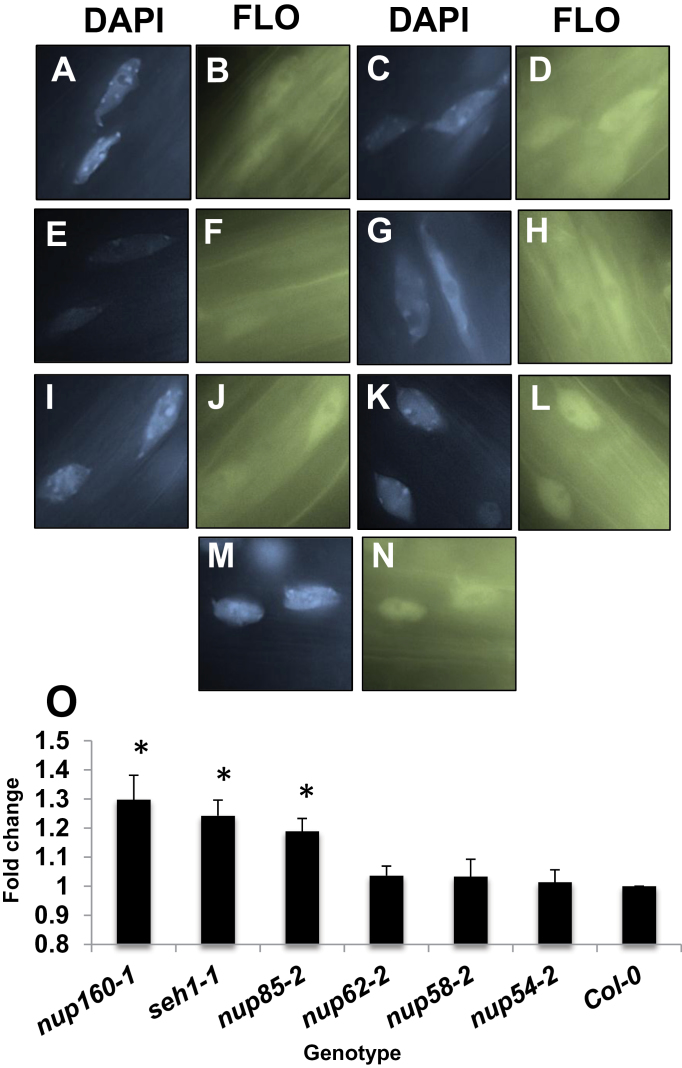
*Nup* mutants exhibit different levels of nuclear mRNA accumulation. Roots from 7-day-old seedlings were treated with an oligo(dT)–fluorescein (FLO) probe and post-stained with DAPI. Representative images A, C, E, G, I, K, and M show nuclei stained with DAPI, while B, D, F, H, J, L, and N show accumulation of FLO in the same nuclei from the following alleles: Col-0 (A, B), *nup62-2* (C, D) *nup54-2* (E, F), *nup58-2* (G, H), *nup85-2* (I, J), *seh1-1* (K, L), *nup160-1* (M, N). FLO accumulation was measured in the nucleus and cytoplasm of root cells from the indicated genotype (>21 cells). The ratio of FLO accumulation between the nucleus and cytoplasm was calculated in each cell and averaged to give a value for each genotype (see Supplementary Fig. S5 at *JXB* online for more detail). In each experiment, this was then expressed as a proportion of the ratio observed in Col-0 cells (in Col-0 cells the FLO ratio was ~1, indicating no nuclear FLO accumulation). This ‘fold change’ was then averaged in four independent experiments (O). Average Student *t*-test values of original values compared with Col-0, *P*<0.03(*). Error bars represent the SE.

Work performed in other eukaryotic systems has demonstrated that cells with altered NPC composition exhibit defects in bulk mRNA nuclear export. This has been observed in *Arabidopsis* as mRNA is preferentially held within the nucleus of certain *nup* mutants (Dong *et al.*, 2006*b*; Parry *et al.*, 2006; Jacob *et al.*, 2007; Xu *et al.*, 2007*b*; Lu *et al.*, 2010; Wiermer *et al.*, 2012; MacGregor *et al.*, 2013). This analysis was extended by quantifying mRNA nuclear accumulation in a new set of *nup* mutants. In this experiment, roots are incubated with a labelled oligo(dT) probe and the fluorescence inside and outside of nuclei was measured in order to quantify the amount of mRNA that has remained within the nucleus (Supplementary Fig. S5 at *JXB* online). Consistent with previous observations, wild-type plants show little nuclear mRNA accumulation as the fluorescent intensity is equivalent inside and outside the nucleus (Fig. 4A, B, O; Supplementary Fig. S5). In *nup160*, *seh1*, and *nup85* nuclei, stronger nuclear fluorescence was quantified that represents nuclear mRNA accumulation (Fig. 4I–O, Supplementary Figs S4, S5). This agrees with the hypothesis that a role of the *Arabidopsis* NUP107–160 complex is to influence mRNA transport. Interestingly although *seh1-1* and *nup85* mutant alleles display a mild defect in mRNA export, their general growth in similar to wild-type plants.

Nuclei from *nup62*, *nup54-2*, and *nup58-2* roots each display levels of mRNA nuclear accumulation equivalent to wild-type nuclei (Fig. 4C–H, O, Supplementary Figs S3, S5 at *JXB* online) despite having significantly altered growth phenotypes. Importantly this is the first time that, when tested, plants with a *nup* mutation do not exhibit a defect in mRNA accumulation. This indicates that the NUP62 subcomplex plays a role distinct from that of other tested NUPs in the control of mRNA nuclear export.

Plants lacking other tested NUPs do show defects in nuclear mRNA export, whether these NUPs are situated within the NUP107–160 complex or lie in peripheral NPC locations (Parry *et al.*, 2006; Jacob *et al.*, 2007; Xu *et al.*, 2007*a*; Lu *et al.*, 2010; Wiermer *et al.*, 2012; MacGregor *et al.*, 2013). Therefore, this may suggest that there is a distinct route of mRNA transport through the plant NPC that does not involve the NUP62 subcomplex.

### Nucleoporin double mutants display a range of growth and molecular phenotypes

Previous work has shown that NUP160 plays an important role in plant NPC function (Fig. 2) (Dong *et al.*, 2006*b*; Parry *et al.*, 2006; Robles *et al.*, 2012). Therefore, to better understand the genetic relationship between NUP160 and other NUPs, certain *nup* mutants were crossed with alleles of *nup160*. These included *nup98a-1*, *nup85-2*, and *nup62-2* plants. In the resulting double mutants a range of phenotypes were observed that highlight the differing genetic relationships of NUP160 with other NUPs.

**Fig. 5.**
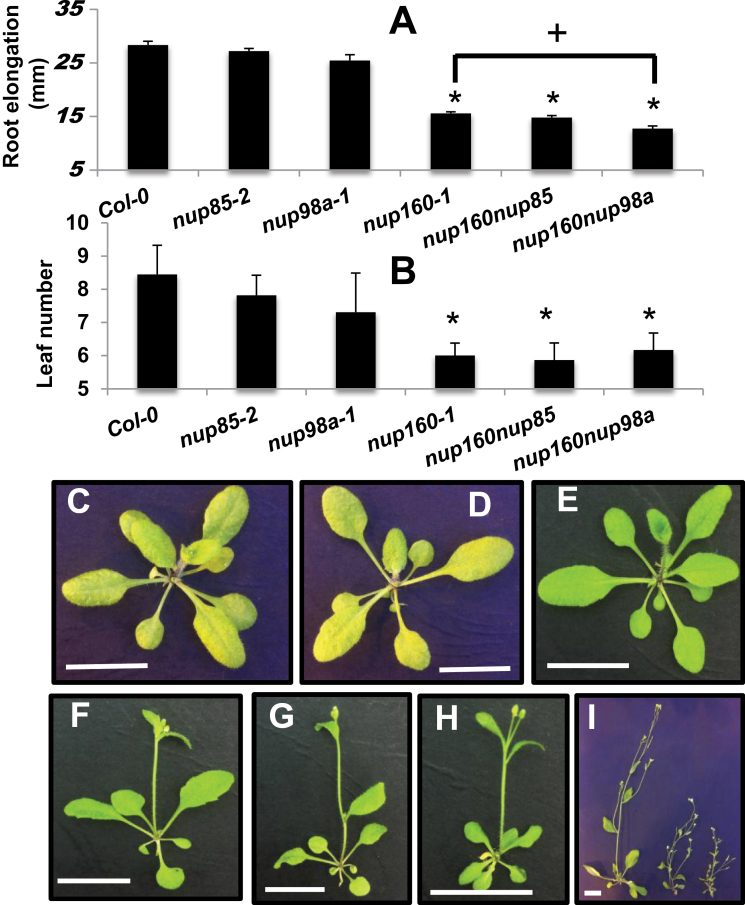
*Nup* double mutants show different phenotypes. Root elongation was measured in light-grown (A) 7-day-old seedlings. Rosette leaf number (B) at the time of flowering in plants grown in LD conditions. Error bars represent the SE. Student *t*-test, compared wiuth Col-0(*) *or nup160-1*(+), **P**<0.01. Representative images of plants grown for 23 d under LD conditions with the following genotypes; Col-0 (C), *nup85-2* (D), *nup98a-1* (E), *nup160-1* (F), *nup160-1nup85-2* (G), *nup160-1nup98a-1* (H). Comparison of 34-day-old plants (I) from L–R: *nup98a-1*, *nup160-1*, *nup98anup160.* Scale bars represent 13mm.

Single *nup160-1* mutants have reduced root elongation and smaller stature, while *nup85-2* plants resemble wild-type plants. The *nup160nup85* double mutant grows similarly to the *nup160* mutant with no increase in phenotypic severity (Fig. 5). This indicates that the loss of NUP160 removes any requirement for NUP85 function and, as discussed below, it is difficult to explain this phenotype with current presumed knowledge of how these proteins are arranged in the NUP107–160 complex (see the Discussion).

NUP98 is a peripheral FG-repeat NUP that in metazoans has multiple functions, not least as a determinant of transport through the NPC (Hulsmann *et al.*, 2012), control of gene expression (Kalverda *et al.*, 2010), and as a potent oncogene (Xu and Powers, 2009). While there is a single NUP98 gene in metazoans, in plants a duplication event (Arabidopsis Genome Consortium, 2000) created both NUP98a and NUP98b genes, where the amino acid sequences are 61% identical. Plants with a T-DNA insertion in each of these genes were identified (Supplementary Fig. S6 at *JXB* online) and it was shown that these plants have wild-type phenotypes (Fig. 5; Supplementary Fig. S6). The T-DNA insertion within *nup98a-1* lies in a 5′ location and appears to prevent transcription of the gene, whereas in *nup98b-2* the insertion lies within the last exon, suggesting that a truncated protein will be produced.

When compared with *nup160-1*, the *nup160nup98a* double mutant has significantly smaller roots and reduced stature (Fig. 5). These more severe phenotypes indicate that the NUP107–160 complex and NUP98 play distinct roles in plant growth. This also suggests that NUP98a performs different functions from NUP98b, as the assumed presence of the latter is unable to make up for the lack of the former. The relationship between these two plant NUP98 proteins are under evaluation and, from current knowledge of the NPC and of NUP98 function in other organisms, it is expected that *nup98anup98b* double mutant plants will not be viable.

Following a cross between *nup160–4* and *nup62-2*, it was not possible to identify viable *nup160-4nup62-2* double mutant plants. This was unsurprising since the *nup62-2* and *nup160-4* single mutants both demonstrate pleiotropic mutant phenotypes and it suggests that the plant is unable to overcome reduced function of both NUP107–160 and NUP62 subcomplexes. The underlying reason for this will become clearer once a better understanding is obtained about the specific cargoes that are differently transported in either of these mutants.

### Gene expression change in nucleoporin-deficient plants

In order to understand the phenotypic changes that are observed in *nup160-4* and *nup62-2* plants, an analysis of global gene expression was performed using the NASCarray service at the Nottingham Arabidopsis Stock Centre.

In the context of the plant NPC, the only previous global expression analysis has investigated either *tpr1* or *hos1* mutant plants. First, post-flowering *tpr1* mutants were compared with pre-flowering wild-type plants and, more recently, early-flowering 14-day-old *hos1* was compared with wild-type plants (Jacob *et al.*, 2007; MacGregor *et al.*, 2013). Interestingly, ~8% of genes showed up-regulated expression in both these experiments. In the case of *hos1*, these included a high proportion of genes under circadian regulation, which reflects the multiple roles that HOS1 appears to play in plant development (MacGregor *et al.*, 2013; Jung *et al.*, 2014).

**Fig. 6.**
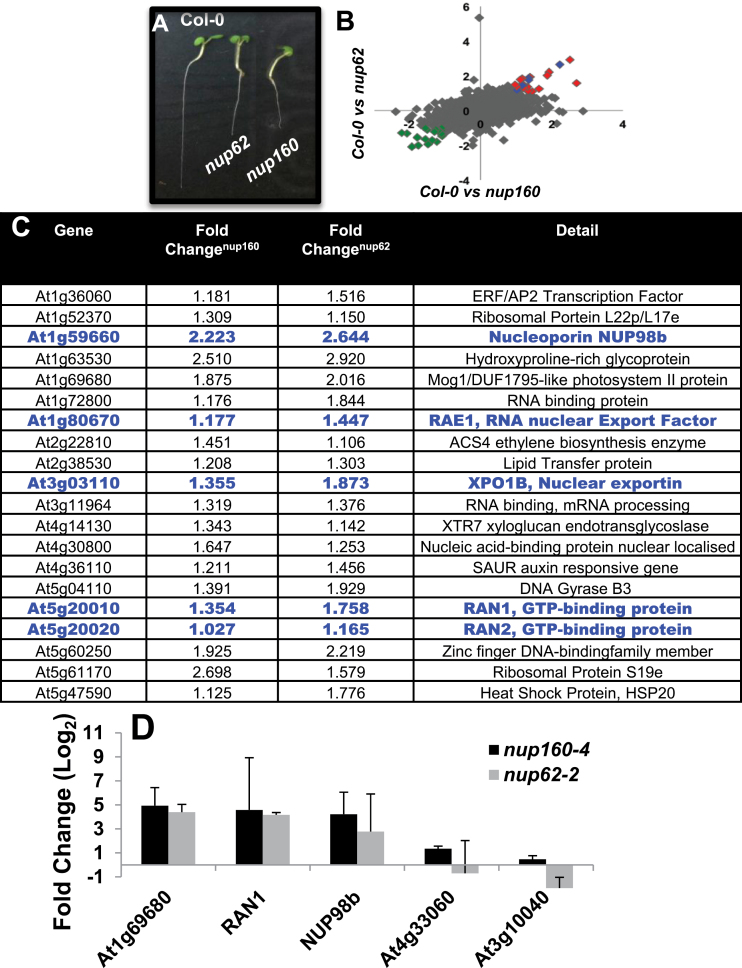
Gene expression change in *nup* mutants. Global gene expression was assessed in 7-day-old Col-0, *nup62*, and *nup160* seedlings (A). Graph (B) shows gene expression changes between Col-0 versus *nup160* (*x*-axis) and Col-0 versus *nup62-2* (*y*-axis, both in log_2_, so 2-fold change=1). Green diamonds represents a 2-fold decrease in both mutants, and red and blue diamonds denote genes with a 2-fold increase which includes 20 annotated genes shown in (C) (ordered by At number). Details of the genes are shown with the fold changes observed in *nup160* and *nup62* seedlings. Genes highlighted in blue and annotated in blue in (B) are thought to be involved in nuclear transport. (D) Results from real-time PCR of expression changes in a selection of genes from (C) or the control genes, At4g33060 and At3g10040. Fold change refers to the ratio in expression change between mutant and wild-type seedlings of the selected genes using Actin7 (At5g09810) as the housekeeping control gene. Bars represent the SE.

In order to avoid the inevitable expression changes that occur during floral transition, gene expression was compared between 7-day-old wild-type, *nup62-2*, and *nup160-4* seedlings (Fig. 6A). Transcript levels from triplicate samples were assessed using the ATH1 Genome Array and, following comparison of average expression levels of wild-type and mutant genes, relatively few genes showed a 2-fold change in gene expression. When *nup160-4* seedlings were compared with the wild type, 159 genes showed 2-fold up-regulation and 174 genes showed similar levels of down-regulation. In *nup62-2*, 76 genes were 2-fold up-regulated and 58 were 2-fold down-regulated. Comparison of these data sets showed that 18 annotated genes were 2-fold down-regulated in both mutants. When the gene ontology (GO) annotations of these genes were analysed, no apparent similarity in their proposed function was revealed (Supplementary Fig. S7 at *JXB* online).

Most notable were the 20 annotated genes that were 2-fold up-regulated in both *nup62-2* and *nup160-4* (Fig. 6B–D). This group includes five genes involved in nuclear transport, namely the nucleoporins NUP98b and RAE1, and the nuclear transport proteins RAN1, RAN2, and XPO1 (Haizel *et al.*, 1997; Blanvillain *et al.*, 2008; Lee *et al.*, 2009; Tamura *et al.*, 2010). Subsequently the expression of RAN1 was assessed in other 7-day-old *nup* mutant seedlings and found to be significantly up-regulated in *nup54-2*, *nup58-2*, *seh1-1*, and *nup85-2* plants (Supplementary Fig. S8 at *JXB* online).

These findings provide an interesting clue to a potential feedback mechanism that may exist to regulate the activity of the plant NPC, namely that in the absence of a particular NUP, expression of other genes involved in nuclear transport is altered. Importantly, if this suggests that a certain level of ‘buffering’ exists in the regulation of nuclear transport components, it may begin to explain why plants appear better able than other eukaryotes to overcome a decline in function of certain NUP proteins.

## Discussion

In this study, the function of a number of *Arabidopsis* NUPs were investigated using phenotypic analysis, cell biological techniques, and measurement of global gene expression.

Although the architecture of the entire NPC is similar across all eukaryotes, the function of individual NUPs can vary greatly in an organism-specific manner. Therefore, nuclear transport will influence plant-specific signalling pathways that respond to phytohormones, biotic and abiotic stresses. It appears that the general phenotypic consequence of removing *Arabidopsis* NUPs takes one of three different forms. First, the plants are unable to survive, as in the case of removing NLP1/CG, NUP205, NUP62, GP210 (Meinke *et al.*, 2008), NUP88 (Wiermer *et al.*, 2010), NUP214, or AtGle1 (Braud *et al.*, 2012). The second group, comprising mutants that mostly produce truncated proteins, have an intermediate phenotype characterized by retarded growth and early flowering, and occurs in plants lacking wild-type function of NUP54, NUP58, NUP62, NUP160, NUP96, NUP136/NUP1, HOS1, and TPR1/NUA (Zhang and Li, 2005; Dong *et al*., 2006*a*, *b*; Parry *et al.*, 2006; Jacob *et al.*, 2007; Xu *et al.*, 2007*b*; Lu *et al.*, 2010; Tamura *et al.*, 2010; Lazaro *et al.*, 2012; Robles *et al.*, 2012; Wiermer *et al.*, 2012; Ferrandez-Ayela *et al.*, 2013; Jung *et al.*, 2013; MacGregor *et al.*, 2013). Finally, reducing the function of certain nucleoporins causes no obvious phenotype, namely NUP98a/b, NUP133, NUP107, NUP85, Seh1, and Sec13 (Wiermer *et al.*, 2012). However certain double mutants can have more severe phenotypes than constituent single mutants, as in the case of *nup160nup62-2*, *nup160nup98a*, or *nup160nup96* (Parry *et al.*, 2006). Overall it appears that NUPs that are not part of NPC subcomplexes are essential for growth, while those within a subcomplex may be functionally ‘buffered’ by the other complex members (Fig. 1). However, gaining a fuller understanding of how these mutants cause their phenotypes requires a greater knowledge of the structural interactions between NUPs. Obtaining this information would enable better predictions as to the effect of any truncated proteins, and is certainly an important task to accomplish as understanding of the plant NPC increases.

Arguably the non-lethal *nup* mutants are most interesting as they provide a platform to understand how the NPC influences different signalling pathways. The data presented here indicate that even though *nup* mutants may share similar phenotypes, the molecular basis for this change is not the same, most notably in the control of nuclear mRNA transport. The mRNA accumulation observed in mutants within the NUP107–160 complex is interesting as it is shown that *seh1-1* and *nup85* seedlings have defective nuclear mRNA accumulation yet do not exhibit major growth defects.

The NUP107–160 complex is a significant structural component of the NPC (Walther *et al.*, 2003; Alber *et al.*, 2007*a*) and, if one can reasonably assume that this structure exists in the plants, then it is surprising that the plant is able to survive with relatively minor phenotypic consequences when the function of members of this complex are reduced, removed, or altered. However, the phenomenon of varied phenotypes that result from changes in different NUP107–160 complex members is somewhat recapitulated across other eukaryotes. In *C. elegans,* reduction of NUP expression by RNA interference (RNAi) shows a wide variation in the amount of embryo lethality, namely NUP107, Seh1 (100% survival), NUP133 (99%), NUP85 (63%), NUP160, and NUP96 (~0%) (Galy *et al.*, 2003).

The function of the NUP107–160 subcomplex is relatively well studied in other organisms. The orthologous yeast NUP84 complex has a Y-shaped structure with the arms of the ‘Y’ formed by NUP120 (orthologous to AtNUP160) and NUP85 (Lutzmann *et al.*, 2002). A more comprehensive dissection of the yeast complex also found that certain members are more important for fitness of the organism, namely both the NUPs lying within the ‘arms’ of the Y-complex and also scNUP145C (orthologous to vertebrate/plant NUP96) (Fernandez-Martinez *et al.*, 2012). Given these seemingly important structural positions, it is difficult to explain why the *nup160nup85* double mutant does not have a more severe growth phenotype than a single *nup160* mutant. This contrasts with the severe growth defects observed in *nup160nup96* double mutants (Parry *et al.*, 2006). Perhaps NUP160 and NUP96 have other functions away from the NPC that are reflected in their more severe phenotypes. Studies across different eukaryotes show that the function of the NUP107–160 complex is determined by a number of key components, although how this corresponds to the structural alignment of the entire NPC remains largely unknown.

Given the limits of the mRNA accumulation assay, it was surprising to discover that members of the NUP62 subcomplex do not appear to participate in mRNA export. This subcomplex sits within the central channel of the NPC yet its role in nuclear transport remains somewhat controversial. One set of largely structural experiments shows that the complex acts as a variable ring allowing transit of molecules through the pore (Solmaz *et al.*, 2011), while a set of transport assays in *Xenopus* oocytes reveals that the complex makes a minimal contribution to passive and active transport (Hulsmann *et al.*, 2012). Currently, this depth of information for the role of the complex in *Arabidopsis* is not available, but it is clear from the lethality of certain *nup62* alleles and the growth phenotypes of mutants in other members that it plays a major functional role. One theory that explains movement of the molecules across the NPC posits that there are distinct paths through the pore (Schoch *et al.*, 2012), and the apparent lack of mRNA accumulation phenotype in the NUP62 complex mutants may suggest that the complex participates in one type of transport pathway but not in all of them. Future investigations into how the complex influences both nuclear import and export will hopefully clarify these roles.

The alteration in nuclear morphology in certain *nup* mutants (Fig 3) mirrors what has been observed in *Arabidopsis nup136-1* plants where nuclear shape is more significantly affected than nuclear size (Tamura *et al.*, 2010). Similar phenotypes are also observed in plants that lack function of inner nuclear membrane-localized SUN proteins (Fig 3), outer nuclear membrane (ONM)-localized WIP proteins, or a novel plant myosin that links the ONM to the actin cytoskeleton (Zhou *et al.*, 2012; Tamura *et al.*, 2013). The recent identification of the KAKU1 myosin protein, which links the ONM and cytoskeleton, begins to explain how these changes in nuclear morphology might occur (Tamura *et al.*, 2013). In wild-type plants, elongated nuclei are observed in leaf epidermal cells as well as in mature root cells, and it is likely that this elongation occurs as a mechanical consequence of an increase in cell volume. As seen in *kaku1* mutants, removing the linkage between the nuclear membrane and cytoskeleton prevents this change in nuclear shape/size and results in more circular nuclei (Tamura and Hara-Nishimura, 2011; Tamura *et al.*, 2013). Therefore, Fig. 3 suggests that the structural NUP107–160 complex of the NPC directly or indirectly interacts with cytoplasmic or nucleoplasmic proteins that help maintain nuclear morphology. During their proteomic analysis of the plant NPC, Tamura and colleagues (Tamura *et al.*, 2010) failed to identify structural proteins of the cytoplasm or nucleoplasm. However, they did not perform immunoprecipitations using NUP160 that was shown in Fig. 3 to have altered morphology, so the possibility remains that these members of the NPC interact with proteins in the direct nuclear periphery.

In experiments to analyse the gene expression changes that result from altered NPC function, surprisingly few genes showed 2-fold changes in either *nup160* or *nup62* mutants (Fig. 6; Supplementary Fig. S7 at *JXB* online). However it is intriguing to discover that genes involved in nuclear transport were up-regulated in both mutants. Performing this type of experiment in other multicellular organisms is difficult due to the general phenotypic severity of *nup* mutants. As a result, there is no published record of this possible feedback between NUP function and expression of genes involved in nuclear transport. Ongoing research at both a global level to investigate nuclear mRNA transport and more precisely looking at individual transcripts will further decipher this relationship, but as it stands these results might indicate that reducing NPC function in *nup* mutants causes a concomitant up-regulation of some genes that may increase the rate of nuclear transport, in order to maintain the status quo and ultimately the viability of the cell. However, in light of these findings, it is difficult to explain why, if there is a compensatory mechanism, certain mutants show strong mutant phenotypes. This probably highlights the complexity of the system where the NPC lies at the nexus of mRNA export and protein import. Any perturbation in the system will undoubtedly lead to numerous cellular changes that amount to pleiotropic phenotypes. However, this again raises the questions as to why some NUPs are more essential than others, and can only be answered with a more targeted study of individual subcomplexes. Although the overall significance of this result remains to be seen, it could have major ramifications regarding our understanding of how the components of the NPC are regulated.

This study highlights the NPC as a central player in the biology of the plant nucleus. Over the coming decade, it is hoped that many of these findings will be extended to gain an understanding as to how the NPC specifically influences plant growth and how this compares with mechanisms of control in other eukaryotes.

## Supplementary data

Supplementary data are available at *JXB* online.

Figure S1. T-DNA insertions and NUP expression.

Figure S2. Growth of *nup* mutants.

Figure S3. Phenotypes of *nup62* mutant plants.

Figure S4. Phenotypes of *nup85* mutants.

Figure S5. mRNA accumulation in *nup* mutants.

Figure S6. Phenotypes of *nup98* mutants.

Figure S7. Gene expression change in *nup* mutants.

Figure S8. RAN1 expression in selected *nup* mutants.

Table S1. Details of *Arabidopsis* nucleoporin mutants.

Table S2. Primer sequences used in mutant characterization or expression analysis.

Supplementary Data
