# Supplementary material for: Components of the Arabidopsis nuclear pore complex play multiple diverse roles in control of plant growth
Source: J Exp Bot. 2014 Aug 27;65(20):6057–67. doi: 10.1093/jxb/eru346 (PMC4203139; doi:10.1093/jxb/eru346)
Supplement: Supplementary Data [file supp_65_20_6057__index.html]

Components of the Arabidopsis nuclear pore complex play multiple diverse roles in control of plant growth — Components of the Arabidopsis nuclear pore complex play multiple diverse roles in control of plant growth — Supplementary Data 

# Components of the *Arabidopsis* nuclear pore complex play multiple diverse roles in control of plant growth

## Supplementary Data

Data files

**Files in this Data Supplement:**

- Supplementary Data - Supplementary Data
