# Supplementary material for: Components of the Arabidopsis nuclear pore complex play multiple diverse roles in control of plant growth
Source: J Exp Bot. 2014 Aug 27;65(20):6057–67. doi: 10.1093/jxb/eru346 (PMC4203139; doi:10.1093/jxb/eru346)
Supplement: Supplementary Data [file supp_eru346_jexbot130393_file001.pdf]

Journal of Experimental Botany
Components of the Arabidopsis Nuclear Pore Complex Play Multiple Diverse Roles in Control of Plant Growth
Geraint Parry

Supplementary Figure Legends

Supplementary Figure 1: T-DNA insertion and NUP expression.

Gene structure, T-DNA insertions (Alonso *et al.*, 2003) and cDNA expression of nucleoporins used in this study; *NUP54 (***A**), *NUP58* (**B**), *SEH1* (**C**), *NUP62* (**D**), *NUP160* (**E**). The expression data suggests that each *nup* mutant produces a truncated protein as outlined in the main text or in previous work (Parry *et al.*, 2006). The left panel shows NUP gene structure (not to scale) with black bars representing exons and black triangles representing approximate T-DNA insertion sites. The lower black line shows position of primers used in right panel. Right panel show RT-PCR using primers from left panel. Primers from Actin7 (At5g09810) were used as an expression control. Sizes of cDNA are as follows: *NUP54*- 1134bp, *NUP58*- 1542bp, *SEH1*- 981bp, *NUP62*- 2220bp, *NUP160*- 4515bp.

Supplementary Figure 2- Growth of *nup* mutants. . Representative 33do plants grown under LD conditions, *Col-0* (**A**), *nup85-2* (**B**), *nup62-2* (**C**), *nup58-2* (**D**), *nup54-2* (**E**), *nup160-4* (**F**). Scale bar represents 30mm. Rosette leaf number (**G**) at the time of flowering of plants growth in SD conditions (12h:12h). Bars represent SD. Students Ttest compared to *Col-0*, P<0.01(*).

Supplementary Figure 3: Phenotype of *nup62* mutant plants. A-

Representative 7do seedlings showing short roots in *nup62* mutants (scale bar-

1cm) or 23 day-old plants (**B**) showing reduced rosette size and early flowering (scale bar- 3cm) grown under LD conditions. mRNA accumulation in nuclei from 7do *Col-0* (**C-D**) or *nup62-1* (**E-F**) roots. See figure 4 and Supplementary Figure S5 for experimental detail. Ratio of amount of FLO-accumulation between Col-0:nup62-1 is 1 : 1.02, n= 60 (*Col-0*) or n= 74 (*nup62-1*). Ttest compared to Col-0, P= 0.44. Scale bar- 10µm.

Supplementary Figure 4: Phenotypes of *nup85* mutants. Gene structure of *NUP85 (***A**) The left panel shows gene structure (*NUP85 cDNA*- 2151bp) with black bars representing exons and black triangles representing approximate T-DNA insertion site. The lower black line shows position of primers used in right panel. Right panel show RT-PCR using primers from left panel. Primers from Actin7 (At5g09810) were used as an expression control. '+' and '-' denote use of RT enzyme during cDNA preparation. (**B**)- Root elongation of 7do seedlings (n≥ 26). (**C**)- Rosette leaf number at the time of flowering when grown under LD (n ≥ 9). Representative pictures of 25do *Col-0* (**D**) or *nup85-3* (**E**) plants. (**F**)- Calculations of mRNA accumulation in wildtype or mutant nuclei from 7do roots as explained in Figure 4 and Supplemental Figure S5. Ratio of amount of FLO-accumulation between *Col-0:nup85-3* is **1:1.14** (n≥ 60, Ttest P< 0.001). Pictures of representative nuclei from *Col-0* (**G-H**) or *nup85-3* (**I-J**) roots. Scale bar- 10µm.

Supplementary Figure 5- mRNA accumulation in *nup* mutants. Low magnification images (10x) of roots from 7do seedlings treated with an oligo(dT)-Fluorescein (FLO) probe and post-stained with DAPI (**A-D**), where nuclei with increase in FLO accumulation are shown in (**C**). (**E**)- FLO accumulation is quantified at higher magnification (63x) by setting single-point measurement positions in images of DAPI-stained nuclei (**E**, **#^{1}, #^{2}**) and then measuring in

identically positioned FLO-image (**E**,**$+^1$.$+^2$**). The pixel intensity is compared between $+^1$ and $+^2$ to give a value for FLO nuclear-accumulation.

To ensure that the background FLO levels in each image are equivalent, five random locations were set in the DAPI image (**E,x^1-x^5**) and the pixel intensity was measured in the equivalent FLO image (**E,z^1-z^5**). For each image these measured pixel intensities were averaged and subsequently a value was obtained for each image file. Pixel intensity was averaged from four independent experiments from each genotype (**F**) and this demonstrated that background fluorescence in the FLO image was not significantly difference in any genotype (Ttest compared to *Col-0*, P> 0.2). Error bars show SE.

Supplementary Figure 6: Phenotypes of *nup98* mutants. Gene structure of *NUP98a (***A**) and *NUP98b* (**B**). The left panel shows NUP gene structure (not to scale) with black bars representing exons and black triangles representing approximate T-DNA insertion site. The lower black line shows position of primers used in right panel. Right panel show RT-PCR using primers from left panel. '+' and '-' denote use of RT enzyme during cDNA preparation. Primers from Actin7 (At5g09810) were used as an expression control. Sizes of cDNA are as follows: *NUP98a*- 3126bp, *NUP98b*- 2994bp. (**C**)- Root length of 7do wildtype and *nup98* mutants showing no significant differences. Rosettes of 20do *Col-0* (**D**), *nup98a-1* (**E**) or *nup98b-2* (**F**) plants grown in LD conditions. Scale bar- 3cm.

Supplementary Figure 7- Gene expression change in *nup* mutants. Global gene expression was assessed in seven day old *Col-0*, *nup62* and *nup160* seedlings. Graphs (**A,B**) showing expression relationship between *Col-0* and *nup160* (**A**) or *nup62* (**B**) seedlings. Position of genes with two-fold expression increase (Log_2) in the mutants are included as highlighted in Fig 6. List (**C**, ordered

by At number) of 18 annotated genes that show two-fold decrease (Log_2) in both *nup62-2* and *nup160-1* as labeled in green in Fig 6. Details of these genes are shown with the fold changes (Log_2) observed in *nup160* and *nup62* seedlings. None of these genes have an apparent link to nuclear transport.

Supplementary Figure 8- RAN1 expression in selected *nup* mutants. Real-time PCR of expression changes in the nuclear transport gene RAN1 (white bars) or control gene At4g33060 (grey bars) in samples taken from 7do seedlings. Fold-change refers to ratio in expression change between mutant:wildtype seedlings of the selected genes using Actin7 (At5g09810) as housekeeping control gene. Bars represent SE obtained from three technical replicates.

Supporting References

Alonso JM, Stepanova AN, Leisse TJ, Kim CJ, Chen H, Shinn P, Stevenson DK, Zimmerman J, Barajas P, Cheuk R, Gadrinab C, Heller C, Jeske A, Koesema E, Meyers CC, Parker H, Prednis L, Ansari Y, Choy N, Deen H, Geralt M, Hazari N, Hom E, Karnes M, Mulholland C, Ndubaku R, Schmidt I, Guzman P, Aguilar-Henonin L, Schmid M, Weigel D, Carter DE, Marchand T, Risseeuw E, Brogden D, Zeko A, Crosby WL, Berry CC, Ecker JR. 2003. Genome-wide insertional mutagenesis of Arabidopsis thaliana. *Science* **301**(5633): 653-657.

Braud C, Zheng W, Xiao W. 2012. LONO1 encoding a nucleoporin is required for embryogenesis and seed viability in arabidopsis. *Plant Physiol* **160**, 823-836.

Ferrandez-Ayela A, Alonso-Peral MM, Sanchez-Garcia AB, Micol-Ponce R, Perez-Perez JM, Micol JL, Ponce MR. 2013. Arabidopsis TRANSCURVATA1 encodes NUP58, a component of the nucleopore central channel. *PLoS One* **8**, e67661.

Meinke D, Muralla R, Sweeney C, Dickerman A. 2008. Identifying essential genes in Arabidopsis thaliana. *Trends Plant Sci* **13**, 483-491.

Parry G, Ward S, Cernac A, Dharmasiri S, Estelle M. 2006. The Arabidopsis SUPPRESSOR OF AUXIN RESISTANCE proteins are nucleoporins with an important role in hormone signaling and development. *Plant Cell* **18**, 1590-1603.

Wiermer M, Cheng YT, Imkampe J, Li M, Wang D, Lipka V, Li X. 2012. Putative members of the Arabidopsis Nup107-160 nuclear pore sub-complex contribute to pathogen defense. *Plant J* **70**, 796-808.

Figure S1

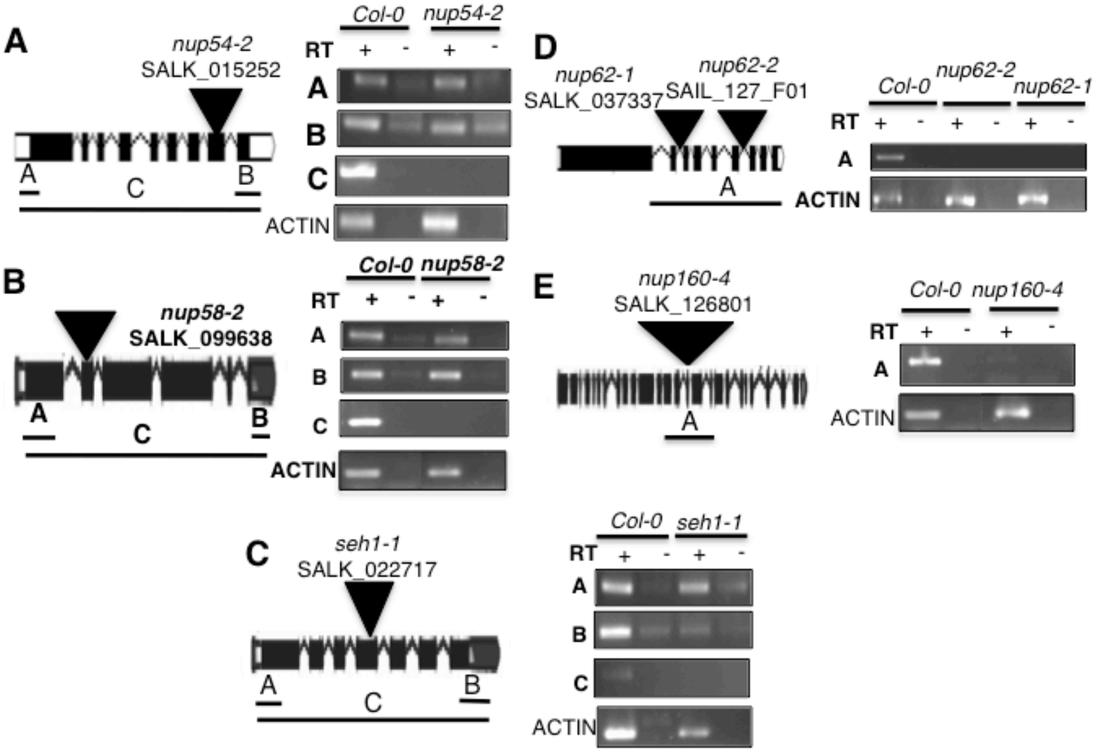

Supplementary Figure 1: T-DNA insertion and NUP expression. Gene structure, T-DNA insertions (Alonso *et al.*, 2003) and cDNA expression of nucleoporins used in this study; *NUP54 (***A**), *NUP58* (**B**), *SEH1* (**C**), *NUP62* (**D**), *NUP160* (**E**). The expression data suggests that each *nup* mutant produces a truncated protein as outlined in the main text or in previous work (Parry *et al.*, 2006). The left panel shows NUP gene structure (not to scale) with black bars representing exons and black triangles representing approximate T-DNA insertion sites. The lower black line shows position of primers used in right panel. Right panel show RT-PCR using primers from left panel. Primers from Actin7 (At5g09810) were used as an expression control. Sizes of cDNA are as follows: *NUP54*- 1134bp, *NUP58*- 1542bp, *SEH1*- 981bp, *NUP62*- 2220bp, *NUP160*- 4515bp.

Figure S2

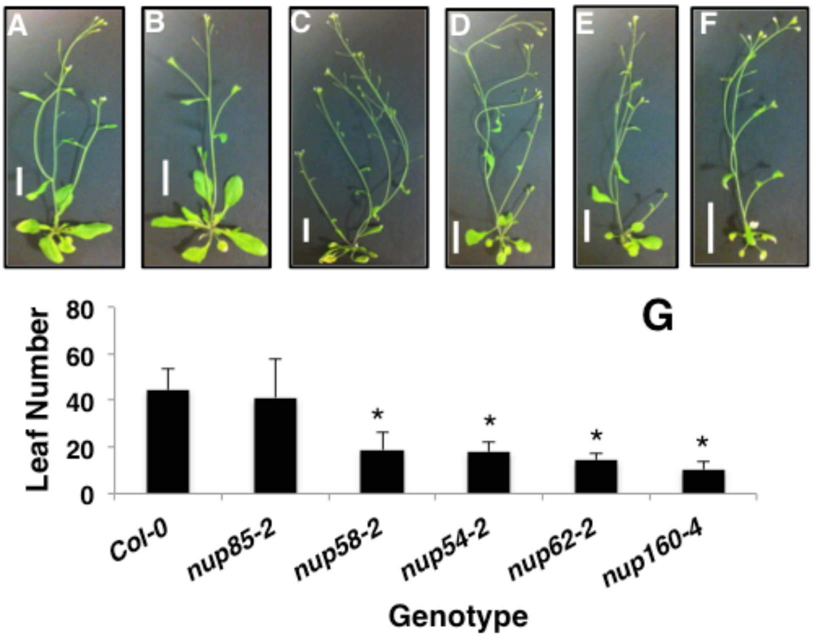

Supplementary Figure 2- Growth of *nup* mutants. Representative 33do plants grown under LD conditions, *Col-0* (**A**), *nup85-2* (**B**), *nup62-2* (**C**), *nup58-2* (**D**), *nup54-2* (**E**), *nup160-4* (**F**). Scale bar represents 30mm. Rosette leaf number (**G**) at the time of flowering of plants growth in SD conditions (12h:12h). Bars represent SD. Students Ttest compared to *Col-0*, P<0.01(*).

Figure S3

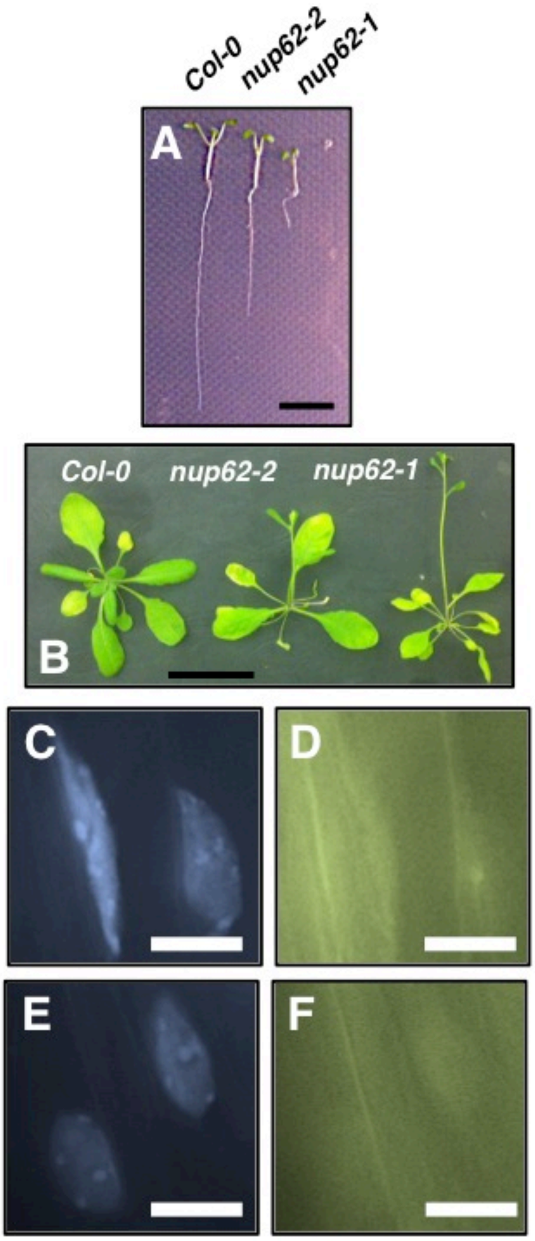

Supplementary Figure 3: Phenotype of *nup62* mutant plants.
A- Representative 7do seedlings showing short roots in *nup62* mutants (scale bar-1cm) or 23 day-old plants (**B**) showing reduced rosette size and early flowering (scale bar- 3cm) grown under LD conditions. mRNA accumulation in nuclei from 7do *Col-0* (**C-D**) or *nup62-1* (**E-F**) roots. See figure 4 and Supplementary Information Fig S5 for experimental detail. Ratio of amount of FLO-accumulation between Col-0:nup62-1 is 1 : 1.02, n= 60 (*Col-0*) or n= 74 (*nup62-1*). Ttest compared to Col-0, P= 0.44. Scale bar- 10μm.

Figure S4

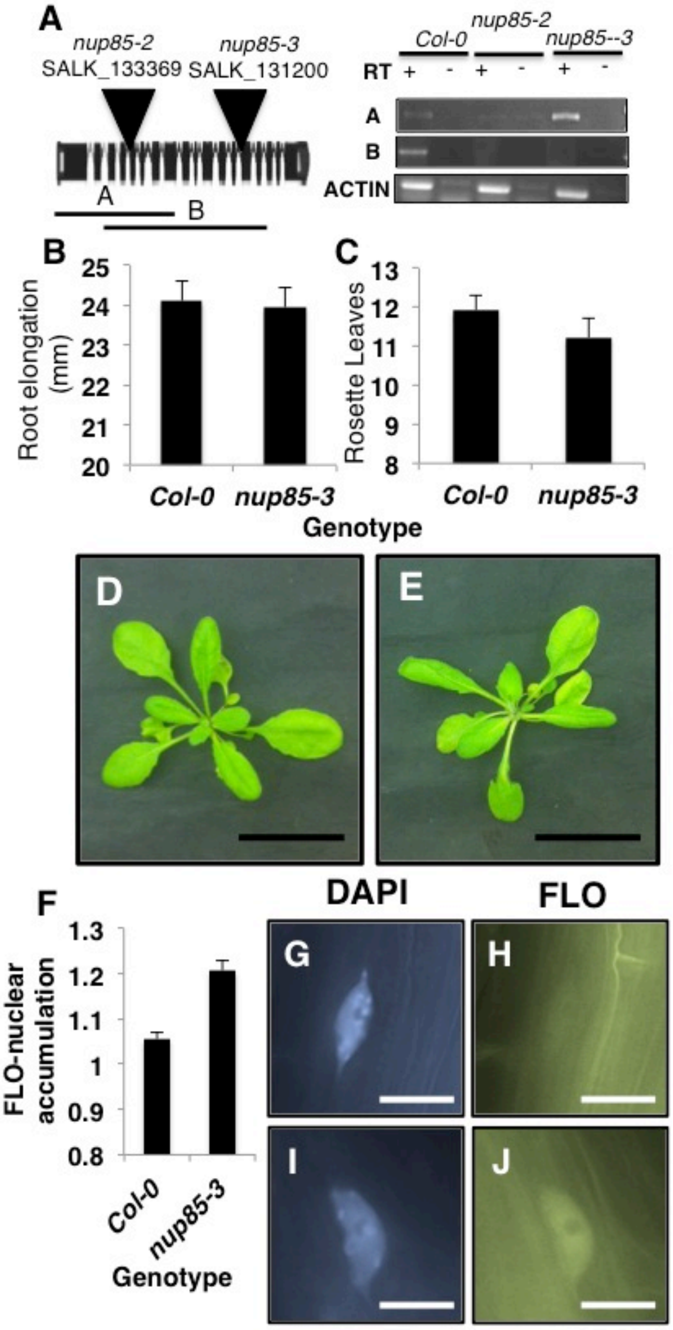

Supplementary Figure 4: Phenotypes of NUP85 mutants.
Gene structure of *NUP85 (***A**) The left panel shows gene structure (*NUP85 cDNA*- 2151bp) with black bars representing exons and black triangles representing approximate T-DNA insertion site. The lower black line shows position of primers used in right panel. Right panel show RT-PCR using primers from left panel. Primers from Actin7 (At5g09810) were used as an expression control. (**B**)- Root elongation of 7do seedlings (n≥ 26). (**C**)- Rosette leaf number at the time of flowering when grown under LD (n ≥ 9). Representative pictures of 25do *Col-0* (**D**) or *nup85-3* (**E**) plants. (**F**)- Calculations of mRNA accumulation in wildtype or mutant nuclei from 7do roots as calculated as explained in Figure 4 and Supplementary Information Fig S5. Ratio of amount of FLO-accumulation between *Col-0:nup85-3* is 1:1.14 (n≥ 60, Ttest P< 0.001). Pictures of representative nuclei from *Col-0* (**G-H**) or *nup85-3* (**I-J**) roots. Scale bar- 10μm.

Figure S5

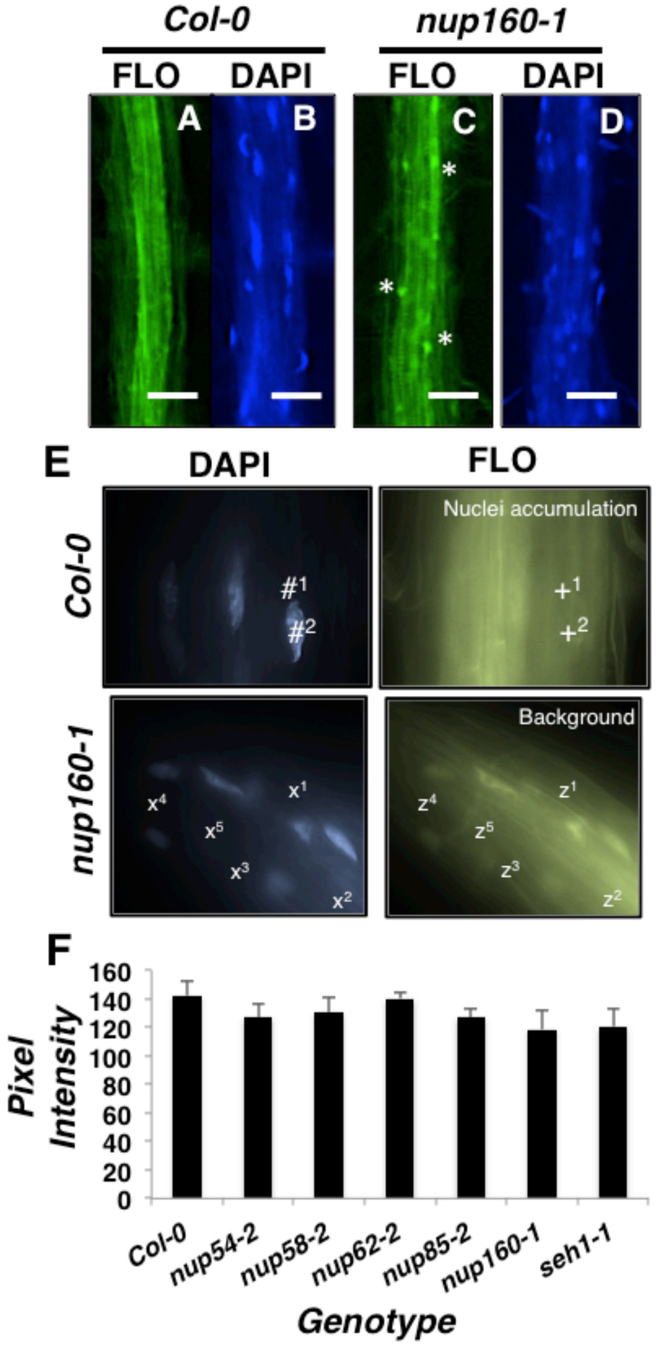

Supplementary Figure 5- mRNA accumulation in *nup* mutants. Low magnification images (10x) of roots from 7do seedlings treated with an oligo(dT)-Fluorescein (FLO) probe and post-stained with DAPI (**A-D**), where nuclei with increase in FLO accumulation are shown in (**C**). (**E**)- FLO accumulation is quantified at higher magnification (63x) by setting single-point measurement positions in images of DAPI-stained nuclei (**E**, **$\#^1$, $\#^2$**) and then measuring in identically positioned FLO-image (**E**,**$+^1$.$+^2$**). The pixel intensity is compared between $+^1$ and $+^2$ to give a value for FLO nuclear-accumulation. To ensure that the background FLO levels in each image are equivalent, five random locations were set in the DAPI image (**E,x^1-x^5**) and the pixel intensity was measured in the equivalent FLO image (**E,z^1-z^5**). For each image these measured pixel intensities were averaged and subsequently a value was obtained for each image file. Pixel intensity was averaged from four independent experiments from each genotype (**F**) and this demonstrated that background fluorescence in the FLO image was not significantly difference in any genotype (Ttest compared to *Col-0*, P> 0.2). Error bars show SE.

Figure S6

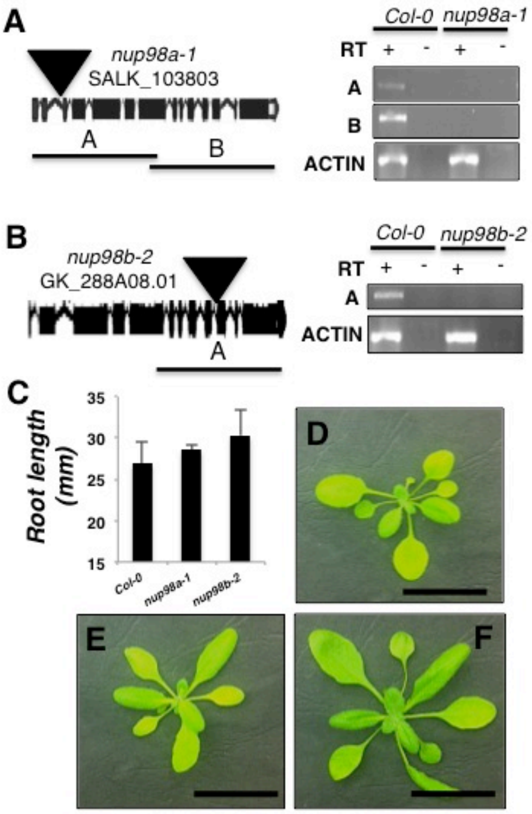

Supplementary Figure 6: Phenotypes of NUP98 mutants. Gene structure of *NUP98a* (**A**) and *NUP98b* (**B**). The left panel shows NUP gene structure (not to scale) with black bars representing exons and black triangles representing approximate T-DNA insertion site. The lower black line shows position of primers used in right panel. Right panel show RT-PCR using primers from left panel. Primers from Actin7 (At5g09810) were used as an expression control. Sizes of cDNA are as follows: *NUP98a*- 3126bp, *NUP98b*- 2994bp. (**C**)- Root length of 7do wildtype and nup98 mutants showing no significant differences. Rosettes of 20do *Col-0* (**D**), *nup98a-1* (**E**) or *nup98b-2* (**F**) plants. Scale bar- 3cm.

Figure S7

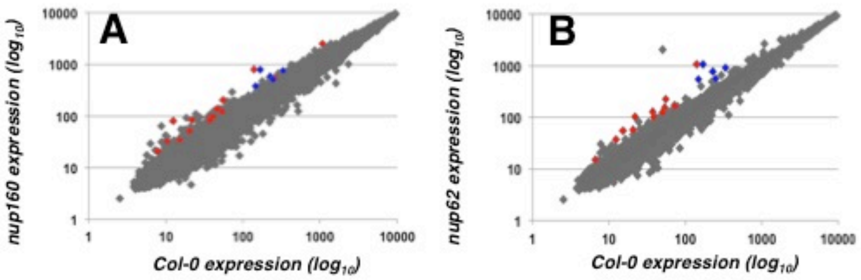

C Gene	Fold Change^{nup160}	Fold Change^{nup62}	Detail
At1g14960	-1.259	-1.076	Polyketide cyclase
At1g52820	-1.772	-1.547	2-oxoglutarate oxygenase
At1g53890	-1.095	-1.130	Unknown protein (DUF567)
At1g66100	-2.070	-1.319	PR-related protein
At1g73010	-1.228	-1.794	PPsPase1
At2g34186	-1.188	-1.188	Unknown protein
At2g34210	-1.099	-1.332	Transcription factor Spt5
At2g43535	-1.169	-1.140	Defensin-like protein
At2g45130	-1.752	-2.012	SPX3 domain protein
At2g45135	-1.553	-1.647	RING/U-box protein
At3g20780	-1.470	-1.935	BIN3, Brassinosteroid signalling
At4g13420	-1.080	-1.459	K+ Transporter
At4g14400	-1.081	-1.079	ACD6, cell death protein
At4g18740	-2.214	-1.284	Rho termination factor
At5g04120	-1.543	-1.007	Phosphoglycerate mutase
At5g36130	-1.502	-1.106	Cytochrome P450
At5g37970	-1.924	-2.087	S-adenosyl-methyltransferase
At5g62160	-1.353	-1.719	Zinc transporter, AtZIP12

Supplementary Figure 7- Gene expression change in *nup* mutants. Global gene expression was assessed in seven day old *Col-0*, *nup62* and *nup160* seedlings. Graphs (**A,B**) showing expression relationship between *Col-0* and *nup160* (**A**) or *nup62* (**B**) seedlings. Position of genes with two-fold expression increase (Log_2) in the mutants are included as highlighted in Fig 6. List (**C**, ordered by At number) of 18 annotated genes that show two-fold decrease (Log_2) in both *nup62-2* and *nup160-1* as labeled in green in Fig 6. Details of these genes are shown with the fold changes (Log_2) observed in *nup160* and *nup62* seedlings. None of these genes have an apparent link to nuclear transport.

Figure S8

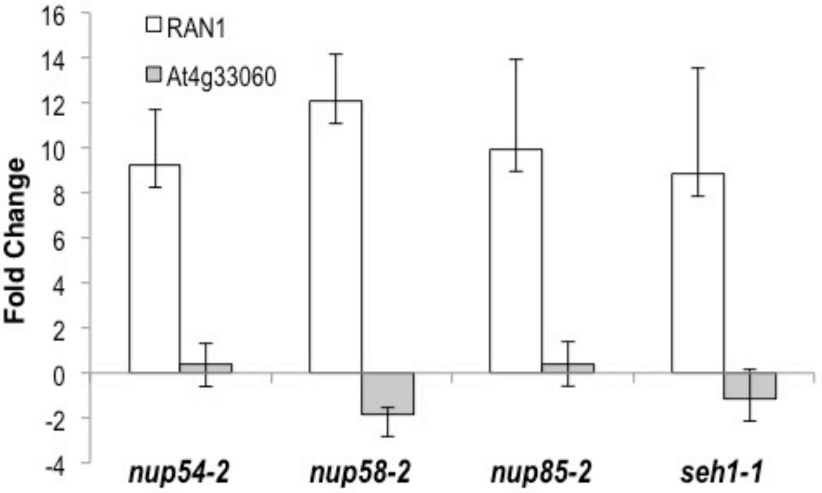

Supplementary Figure 8- RAN1 expression in selected *nup* mutants. Real-time PCR of expression changes in the nuclear transport gene RAN1 (white bars) or control gene At4g33060 (grey bars) in samples taken from 7do seedlings. Fold-change refers to ratio in expression change between mutant:wildtype seedlings of the selected genes using Actin7 (At5g09810) as housekeeping control gene. Bars represent SE obtained from three technical replicates.

Supplementary Table 1: Details of *Arabidopsis* nucleoporin mutants

Nucleoporin	Allele	Mutant Type	mRNA	Embryo	Root Length	Flower-ing Time	Note
NLP1/CG1-At1g75340	*Nlp1-1*	Salk_006526	-	Lethal	-	-	This study
NUP54-At1g24310	*Nup54-2*	Salk_015252	TC	Normal	Short	Early	This study Ferrandez-Ayela et al (2013)[a]
NUP58-At4g37130	*Nup58-2*	Salk_099638	TC	Normal	Short	Early	This study Ferrandez-Ayela et al (2013)[a]
NUP62-At4g37130	*Nup62-1*	Salk_037337	TC	Normal	Short	Early	This study Ferrandez-Ayela et al (2013)[a]
NUP62-At4g37130	*Nup62-2*	Sail_127_F01	TC	Normal	Short	Early	This study Ferrandez-Ayela et al (2013)[a]
NUP62-At4g37130	*Nup62-3*	Sail_1168_C12	Null	Lethal	-	-	EMB2766[b,c]
NUP62-At4g37130	*Nup62-4*	Salk_071521	Null	Lethal	-	-	This study
NUP85-At4g32910	*Nup85-2*	Salk_133369	Null	Normal	WT	WT	This study
NUP85-At4g32910	*Nup85-3*	Salk_131200	TC	Normal	WT	WT	This study
NUP96-At1g80680	*Nup96-1*	Point mutation	Normal	Normal	Short	Early	Parry *et al* (1996)[d]
NUP96-At1g80680	*Nup96-3*	Salk_109959	TC	Normal	Short	Early	Parry *et al* (1996)[d]
NUP98a At1g10390	*Nup98a-1*	Salk_103803	Null	Normal	WT	WT	This study
NUP98b At1g59660	*Nup98b-2*	GK_288A08.01	TC	Normal	WT	WT	This study
NUP160 At1g33410	*Nup160-1*	Point mutation	Normal	Normal	Short	Early	Parry et al (1996)[d]
NUP160 At1g33410	*Nup160-4*	Salk_126801	TC	Normal	Short	Early	This study Parry *et al* (1996)[d]
NUP205 At5g51200	*Nup205-1*	Salk_055559	-	Lethal	-	-	EMB3142[b,e]
NUP205 At5g51200	*Nup205-2*	Sail_874_A02	-	Lethal	-	-	This study
NUP214 At1g55540	*Nup214-1*	Sail_222_E10	-	Lethal	-	-	Braud *et al* (2012)[f]
NUP214 At1g55540	*Nup214-3*	Sail_220_H11	-	Lethal	-	-	This study
Seh1 At1g64350	*Seh1-1*	Salk_022717	TC	Normal	WT	WT	This study Wiermer *et al* (2012)[g]

From L-R columns include- Name of nucleoporin; Allele ID; Type of Mutant T-DNA insertion; mRNA expression in allele (Fig. S1), dash indicates inability to assess mRNA in homozygous mutant, KD- knockdown, TC- probable truncated mRNA; Embryo phenotype, 'lethal' indicates inability to isolate homozygous mutant plants; Root length compared to *Col-0* (WT) seedlings at 7dpg (Figure 2); Time to first flowering in LD conditions compared to *Col-0* (WT) plants (Fig. 2b); Notes, superscript numbers a- Ferrandez-Ayela *et al* (2013) (Ferrandez-Ayela *et al.*, 2013) b- Meinke *et al*, 2008 (Meinke *et al.*, 2008) c- http://www.seedgenes.org/SeedGeneProfile?geneSymbol=EMB+2766 d- Parry *et al*, 2006 (Parry *et al.*, 2006) e- http://www.seedgenes.org/SeedGeneProfile?geneSymbol=EMB+3142 f- Braud *et al*, 2012 (Braud *et al.*, 2012) g- Wiermer *et al*, 2012 (Wiermer *et al.*, 2012)

Supplementary Table 2- Primer sequences used in mutant characterization or expression analysis (Fig 6, Supporting Figure S2).

ALLELE	Forward Primer	Reverse Primer
nup54 A	ATGTTCGGCACTCCGTCTTCATCGC	GATGGCGTACCGAACGCAGGC
nup54 B	CAGAGCTTGATAGATATGCAGG	TGAGTCTAGTGCCATTTCTG
nup54 C	ATGTTCGGCACTCCGTCTTCATCGC	TGAGTCTAGTGCCATTTCTG
nup58 A	CACCATGTCGTTTTTTCCCCCACAGC	TTGCTGCGGCTGCGATTGCG
nup58 B	GGCCAGACAACTCCATCAC	ACGGCGTGTAGTTCGAGATTTTGG
nup58 C	CACCATGTCGTTTTTTCCCCCACAGC	ACGGCGTGTAGTTCGAGATTTTGG
nup62 A	CCTGCTACTAGTTCTGCTAC	AGACATCCAGTGCTTTGGAGC
nup85 A	ATGCCGGGTATGTCTTCGGAATCTGG	CCCATCTTCTAGTTGTGGCC
nup85 B	CTCCTTTCTAGCTCACATCC	CCCATGTTTCCAATGATGC
nup98a A	CACCGCTTGGCCATCCGTTGTCAAACTACA GAGGAAGATG	AGCCAAATCCAGGAGTGC
nup98a B	GTTCATCAAGCACTCCTGG	CACACAACAAAGGAGTAGAGCAC
nup98b A	CATTTGCACAAAATACCACTCC	GACAACGACTTTGGTCCTTTTCTA
nup160 A	GGTGAAGGATCTTGGAAGGC	GCCGATGAAAGGTAGATGTCC
seh1 A	GCTCTTGAATCCATTGGAGC	GGAGGGAACTGGTTCAAGCG
seh1 B	GCTCTTGAATCCATTGGAGC	CGAAGAAGACGAAGTAGAGG
seh1 C	GGAATGGGACATGAGTGGC	GGAGGGAACTGGTTCAAGCG
**MUTANT**	**Forward Primer**	**Reverse Primer**
nlp1-1	CACCATGAGGAAGGAACTGTGTAG	TTGTGTACCAAAGCCAAAGGG
nup205-1	CTTCTGATCCGCTTGATGG	CAGCGGACGCCAAACATCGG
nup205-2	GTCAGCTCCAGAGCAGAGG	GCAACAGCAATGTTGGTTGGC
nup214-1	CACCATGAGCAGAGTTGAGATTGAA	GTTCATCATCTCCGGAACGG
nup214-3	GTGGTTTCAAGGATTCTTGCGC	CTCAGGATTCAACTTCTGGC
**GENE**	**Forward Primer**	**Reverse Primer**
At1g69680	CAGGAACTGTTACCATTGTCGC	GATGAAGAACTTTGAACTGG
RAN1 (At5g20010)	GTGAAGAACAGGCAAGTG	CTGTAAGAGCTCGGCCTCG
NUP98b (A15g59960)	ATGTTCGGTTCTTCTAATAATAATC	GAACTCCCTGTCTGTGCGCC
NTF2 (At1g27970)	ATGTCTCAGATGGATCCCGACGC	GGCGAGCTGGAGATTACCGG
At4g33060	ATGGACACCAAAATCGGATC	GGTTGAGGTCCGGCTCAGCC
At3g10040	ATGGAATCGAATGTGATGTTC	GCCTAACCATTGTATCCGTCC
ACTIN7 (At5g09810)	ATGGCCGATGGTGAGGATATTCAGC	CCAACCATGACACCAGTGTGCC
